# Está na Hora de Avançarmos do Diagnóstico para a Intervenção Ativa nos Ambientes de Trabalho Brasileiros? Um Apelo para Ação

**DOI:** 10.36660/abc.20210619

**Published:** 2021-09-01

**Affiliations:** 1 Universidade de São Paulo São Paulo SP Brasil Universidade de São Paulo (USP), São Paulo, SP – Brasil; 2 Hospital das Clínicas da Faculdade São Paulo SP Brasil Hospital das Clínicas da Faculdade de Medicina de Ribeirão Preto - Cardiologia, São Paulo, SP - Brasil

**Keywords:** Hipertensão, Saúde do Trabalhador, Epidemiologia

A hipertensão arterial (HA) é um dos principais fatores de risco de insuficiência cardíaca, infarto do miocárdio, acidente vascular cerebral (AVC) e doença renal crônica.^1^ De acordo com a Pesquisa Nacional de Saúde de 2013, 21,4% (IC95% 20,8-22,0) dos adultos autorrelataram a presença de HA; no entanto, considerando as medições da pressão arterial (PA) dos respondentes e o uso de medicação anti-hipertensiva, a taxa de adultos com PA maior ou igual a 140/90 mmHg chegou a 32,3% (IC95% 31,7-33,0). A prevalência de HA foi maior entre os homens, e, conforme o esperado, essa taxa foi altamente influenciada pela idade, chegando a 71,7% dos indivíduos com mais de 70 anos de idade.^2^^,^^3^

Portanto, todos os estudos que determinam a prevalência de HA são muito importantes para programas de prevenção, tratamento e acompanhamento. As amostras representativas da força de trabalho são de especial importância, pois incluem a população mais jovem, em idade produtiva e com maior expectativa de vida.

Diversos estudos publicados no Brasil^4^–^6^ avaliaram a prevalência de HA e outros fatores de risco em populações de trabalhadores industriais de diferentes áreas de especialização. Nesses estudos, a prevalência de HA variou de 6% (estágio 2), de acordo com o *VII Joint National Committee*,^7^ a 19%-35,1% em diferentes regiões brasileiras entre adultos de diferentes idades (idade média de 35,4 anos).^8^

Já está bem estabelecido que diversos fatores de risco contribuem para o desenvolvimento da HA, como obesidade, abuso de álcool, sedentarismo, hereditariedade, idade, sexo, entre outros.^2^

No artigo de Xavier et al.,^9^ publicado neste volume dos Arquivos Brasileiros de Cardiologia, os autores relataram a prevalência de HA em uma população de trabalhadores industriais no Rio Grande do Sul, Brasil, de pelo menos cinco setores de atividades industriais distintas. Os autores também avaliaram os fatores de risco para o desenvolvimento de HA. O achado de prevalência média de 10,3% em pessoas com idade média de 32,8 anos está de acordo com o observado anteriormente.

Em relação aos fatores determinantes de HA, foram observadas relações diretas entre idade e PA elevada, sendo mais frequente em homens do que mulheres, em pessoas com excesso de peso e obesas e naqueles com baixa escolaridade e hereditariedade para hipertensão. Esses dados são compatíveis com os relatados em estudos anteriores e em diretrizes de HA.

Em relação ao estudo, questões metodológicas podem afetar alguns dos achados, principalmente a ausência de correlações estatisticamente significativas com renda familiar, tabagismo, consumo de álcool e nível de atividade física, mesmo considerando que os dados foram obtidos há mais de uma década.

Primeiro: o método auscultatório de medição da PA está certamente mais sujeito a erros metodológicos e requer equipe qualificada para ser consistente. Hoje em dia, equipamentos automáticos bem validados são preferíveis para o diagnóstico, conforme recomendado por diretrizes recentes.^2^

Segundo: a análise do estado nutricional por meio do índice de massa corporal (IMC) pode ser refinada com a medição da circunferência da cintura, que está mais relacionada ao prognóstico, elimina a imprecisão do IMC na estimativa do acúmulo de gordura^10^ e aparenta ser mais precisa em relação à HA.^11^

Em resumo, o estudo expõe fatores que podem contribuir para a incidência da HA em uma determinada população de trabalhadores industriais, alertando para a necessidade de prevenção, diagnóstico e orientação para uma doença de alta morbidade que contribui para altas taxas de mortalidade. Ainda, reproduz dados anteriores e leva a um questionamento intrigante: está na hora de avançarmos para além do diagnóstico?

No momento atual, pode ser apropriado utilizar os grandes bancos de dados de todas as regiões do país para dar um passo adiante e propor intervenções ativas lideradas por organizações de apoio já existentes e organizadas (SESI e SENAI, por exemplo). Medidas simples, como atividades de promoção da saúde no ambiente de trabalho,^12^ o planejamento de alimentação saudável para indústrias com refeitório e até a oferta gratuita de lanches saudáveis, podem impactar futuramente os fatores de risco para HA e sua modificação ou, pelo menos, a percepção individual de bem-estar.^13^ Algumas iniciativas em nosso país têm apresentado bons resultados e precisam ser ampliadas.^14^ A Sociedade Brasileira de Cardiologia deve liderar os esforços nessa direção!
