# Supplementary material for: Está na Hora de Avançarmos do Diagnóstico para a Intervenção Ativa nos Ambientes de Trabalho Brasileiros? Um Apelo para Ação
Source: Arq Bras Cardiol. 2021 Sep 1;117(3):492–3. [Article in Portuguese] doi: 10.36660/abc.20210619 (PMC8462962; doi:10.36660/abc.20210619)
Supplement: Translation

# Translation: Is It Time to Move From Diagnosis to Active Intervention in Brazilian Workplaces? A Call for Action

**Affiliations:** 1 São Paulo SP Brazil Universidade de São Paulo (USP), São Paulo, SP – Brazil; 2 São Paulo SP Brazil Hospital das Clínicas da Faculdade de Medicina de Ribeirão Preto - Cardiologia, São Paulo, SP - Brazil

**Keywords:** Hypertension, Occupational Health, Epidemiology

Arterial hypertension (AH) is a major contributing risk factor for heart failure, myocardial infarction, stroke, and chronic kidney disease.^1^ According to the 2013 Brazilian National Health Survey, 21.4% (95%CI 20.8-22.0) of adults self-reported having AH; however, considering their blood pressure (BP) measurements and the use of antihypertensive medications, the rate of adults with BP greater than or equal to 140/90 mm Hg reached 32.3% (95% CI 31.7-33.0). The prevalence of AH was higher among men, and, as expected, this rate was highly affected by aging, reaching 71.7% for individuals over 70 years of age.^2^^,^^3^

Therefore, every study to determine the prevalence of AH is very important for prevention, treatment, and follow-up programs. Samples representing the workforce are of particular importance because they are younger, in the productive age and have a longer life expectancy.

Several studies published in Brazil^4^–^6^ have assessed the prevalence of AH and other risk factors in populations of industrial workers across different areas of expertise. In these studies, the prevalence of AH ranged from 6% (stage 2), according to the VII Joint National Committee,^7^ to 19%-35.1% in different Brazilians regions among adults of different ages (mean age of 35.4 years).^8^

It is well established that several risk factors contribute to the development of AH, such as obesity, alcohol abuse, physical inactivity, heredity, age, and sex, among others.^2^

In the article by Paula Brustolin Xavier et al.,^9^ published in this issue of the *Arquivos Brasileiros de Cardiologia,* the authors report the prevalence of AH in a population of industrial workers in Rio Grande do Sul, Brazil, representing at least 5 sectors of distinct industrial activities. They also assess the risk factors for the development of AH. The finding of a mean prevalence of 10.3% in people with a mean age of 32.8 years is in agreement with those previously observed.

As for the determining factors of AH, direct relationships have been observed between age and higher BP, being more frequent in men than in women, in overweight and obese people, and in those with low education and heredity for hypertension. These data are compatible with those reported in previous studies and guidelines on AH.

Regarding their work, methodological issues may affect some of the findings, especially the absence of statistically significant correlations with family income, smoking, alcohol consumption, and level of physical activity, even considering that they were obtained more than a decade ago.

First: The auscultatory method of measuring BP is certainly more prone to methodological errors and requires well-trained personnel to be consistent. Nowadays, well-validated automatic equipment is preferable for diagnosis, as recommended by recent guidelines.^2^

Second: The analysis of nutritional status using body mass index (BMI) can be refined with the measurement of waist circumference, which is better related to prognosis, eliminates the imprecision of BMI in estimating fat accumulation,^10^ and seems to be more precise in relation to AH.^11^

In summary, the study sheds light on factors that can contribute to the incidence of AH in a defined population of industrial workers and, as such, alerts to the need for prevention, diagnosis, and guidance for a disease with high morbidity that contributes to high mortality rates. It reproduces previous data and leads to an intriguing question: Is it time to move forward from diagnosis?

At the present time, it may be appropriate to use the large databases from all regions of our country to take a step further and propose some active interventions led by the existing well-organized support organizations (SESI and SENAI, for example). Simple measures, such as workplace health promoting activities,^12^ healthy meal planning for industries with a cafeteria, and even the offer of free healthy snacks, may have a future impact on risk factors for AH and their modification or, at least, on the personal perception of well-being.^13^ Some initiatives in our country have produced good results and need to be expanded.^14^ The Brazilian Society of Cardiology must lead this effort!!
